# The Identification and Referral to Improve Safety Programme and the Prevention of Intimate Partner Violence

**DOI:** 10.3390/ijerph18115653

**Published:** 2021-05-25

**Authors:** Amir Reza Akbari, Benyamin Alam, Ahmed Ageed, Cheuk Yin Tse, Andrew Henry

**Affiliations:** 1 Medical School, The University of Manchester, Oxford Road, Manchester M13 9PL, UK; benyamin.alam@student.manchester.ac.uk (B.A.); ahmed.ageed@student.manchester.ac.uk (A.A.); cheuk.tse-2@student.manchester.ac.uk (C.Y.T.); 2 Trauma & Orthopaedics, Salford Royal NHS Foundation Trust, Salford M6 8HD, UK; andrew.henry@srft.nhs.uk

**Keywords:** intimate partner violence, identification and referral to improve safety, sexual health, domestic violence and abuse, public health, fracture clinic

## Abstract

*Introduction:* Intimate Partner Violence (IPV) is a global epidemic which 30% of women experience world-wide. Domestic violence has serious health consequences, with an estimated cost of 1.7 billion annually to the NHS. However, healthcare professionals remain uncertain on how to manage IPV. In 2007, the Identification and Referral to Improve Safety (IRIS) was introduced within primary care to address this shortcoming. The aim of this project is to analyse the impact of IRIS, whilst discussing the extension into secondary care. *Materials and Methods:* A literature review was conducted using PubMed, Cochrane Library and Google scholar. The official IRIS publication list for randomized controlled trial data. *Results:* General practices with IRIS displayed a threefold increase in the identification of IPV and sevenfold increase in referrals. IRIS is cost-effective and under the NICE threshold of £20,000 per quality-adjusted life year gained. Additionally, a systematic review illustrated that one in six women presenting to the fracture clinic experienced IPV within the last year. *Conclusions:* The implementation of IRIS into general practice proved to be cost-effective. Orthopaedic fracture clinics are at the forefront of dealing with IPV, and therefore an adapted IRIS programme within this setting has potential in the prevention of IPV.

## 1. Introduction

### 1.1. Background

Intimate Partner Violence (IPV) is one of the most common forms of family violence, along with Domestic Violence and Abuse (DVA) [1]. It includes multiple types of physical, psychological and sexual abuse, such as physical violence, threats, intimidation, isolation, emotional abuse, economic coercion, manipulation and the assertion of privilege [1]. IPV has been found to be a widespread phenomenon in every country around the world [2]. IPV occurs in almost all settings and effects people regardless of race, ethnicity, class, religious belief, age, immigration status and ability [2]. Even in developed counties such as the UK, the problem is so common and widespread that it has almost become an invisible form of crime and an everyday story in many households [2].

DVA has serious health consequences which have been the subject of numerous studies [3]. DVA is a significant risk factor for many physical and psychological health problems frequently encountered in primary care setting in UK [4]. It has been estimated that the cost associated with DVA to NHS is £1.7 billion per year, with the major cost borne by acute trusts and primary care [5].

The healthcare setting therefore offers a critical and unique opportunity for early identification and prevention of abuse [6]. The professionals from every aspect of our healthcare setting have daily contact with patients and are in a privileged position to help victims. These are patients whose health is damaged by domestic violence and are subsequently very likely to face extreme mental and physical injuries [3]. However, it appears that most healthcare professionals are uncertain on how to deal with domestic abuse victims [7]. While the guidelines from the National Institute for Health and Care Excellence (NICE) now recommend that there should be training around domestic violence at every level, it remains minimal or absent in most medical schools [8]. 

The principal objective of this study was to review the available literature and present the findings regarding a new training and support programme called the IRIS. Identification and Referral to Improve Safety (IRIS) is a training and support programme to improve the response to DVA in general practice [9]. IRIS was initially carried out as a pragmatic cluster-randomised trial, but it has now been implemented in over 35 administrative localities in the UK [10]. The trial and local evaluations of the IRIS implementation showed an increase in patient referrals from general practice to specialist domestic violence agencies [7]. In the final part of this report, an attempt will be made to conduct a feasibility study with regard to introducing IRIS to secondary care. 

However, before discussing the IRIS programme and the prevention of IPV, it is important to recognize the causes of domestic violence, different forms and definitions, the background and scale of the problem, the impact on society, and the role of health care professionals.

### 1.2. Definitions

The terms such as violence against women, domestic violence, domestic abuse, intimate partner violence and gender-based violence are often used without noticing that there are in fact some subtle differences in what each term describes. It is therefore beneficial to consider a brief description of these terms.

Domestic violence: ‘Domestic violence is considered to be all acts of physical, sexual, psychological or economic violence that occur within the family or domestic unit or between former or current spouses or partners, whether or not the perpetrator shares or has shared the same residence with the victim’ [11]. 

Violence against women: ‘Violence against women is understood as a violation of human rights and a form of discrimination against women and shall mean all acts of gender-based violence that result in, or are likely to result in, physical, sexual, psychological or economic harm or suffering to women, including threats of such acts, coercion or arbitrary deprivation of liberty, whether occurring in public or in private life’ [11].

Gender-based violence: It is recognised that gender-based violence is violence that is directed at an individual based on their biological sex, gender identity or perceived adherence to socially defined norms of masculinity and femininity [12]. The United Nations Declaration on the Elimination of Violence against Women (1993) describes violence against women as: ‘any act of gender-based violence that results in, or is likely to result in, physical, sexual or psychological harm or suffering to women, including threats of such acts, coercion or arbitrary deprivation of liberty, whether occurring in public or in private life’ [12]. This incorporates many forms of abuse that occur throughout the life cycle, including within the family and within communities [12]. The types of gender-based violence include: rape and sexual assault; sexual harassment and intimidation at work and other settings; childhood sexual abuse; domestic abuse; stalking; harmful traditional practices such as early and forced marriage; so-called ‘honour’ based violence and female genital mutilation; sex trafficking; and commercial sexual exploitation [12].

Intimate Partner Violence (IPV): The World Health Organisation defines Intimate Partner Violence (IPV) as a ‘behaviour by an intimate partner or ex-partner that causes physical, sexual or psychological harm, including physical aggression, sexual coercion, and psychological abuse and controlling behaviours’ [1]. The concept of physical and sexual abuse is easily defined but psychological harm has a much broader definition which includes coercive and controlling behaviour. Coercive behaviour is a type of abuse which includes threats, blackmailing, humiliation and intimidation [13]. This behaviour is intended to harm, punish or frighten the victim [13]. Controlling behaviour is a range of acts designed to make the victim subordinate and isolate them from sources of support [13]. This enables the perpetrator to exploit their resources and capacities for personal gain by depriving them of independence and escape [13].

The study presented here focuses specifically on domestic abuse against women when the perpetrator is a current or previous partner. The terms Intimate Partner Violence (IPV) and Domestic Violence Abuse (DVA) are therefore used in this context throughout this report.

### 1.3. Scale of the Problem

Numerous studies have reported IPV to be a global problem in developing and developed countries. In order to have a better understanding of the degree and severity of the problem, it is worth considering some of the published data with regards to the level of intimate partner violence recorded around the world. It is reported that:

- Globally the lifetime prevalence of physical and sexual intimate partner violence and abuse for women is around 30% [14].
- In the United States findings from the 1998 National Violence Against Women Survey showed that 1.5 million women are raped or physically assaulted by an intimate partner annually [15]. In addition, only 36% of the women injured during their most recent rape and 30% of the women injured during their most recent physical assault received some type of medical treatment [15]. Other studies found an incidence of battering from 7% to 44%, depending on the sampled population [15].
- Across Europe, an average of 22% of women report experiencing physical and/or sexual violence and that 43% have experienced psychological abuse from the age of 15 [16]. A total of 58% of women across Europe who did not feel that they had an equal say in household finances experienced psychological abuse compared to 22% of women who believed that they had an equal say [16].
- In the UK, the reported rate of physical and/or sexual violence was 29% and psychological abuse 46% [17]. Out of the reported crimes, 25% of women had reported the most serious incident of intimate partner violence and abuse [17]. However, women are more likely to contact healthcare services about the most serious incident of abuse they had experienced [17].
- In Scotland in 2018/19, 60,641 incidents were reported to police [18]. Of these, 82% were reported as a female victim and male perpetrator [18]. In contrast, male victims and female perpertrator accounted for 16% of cases [18].
- In England and Wales between 2019/2020 the crime survery estimates that 2.3 million adults aged 16–74 years experienced domestic abuse [19].

It is a well-established fact that IPV cuts across all levels of society, social divisions and can affect everyone [2]. Furthermore, acts of violence against women are not isolated events but rather patterns of behaviour that violate their human rights [2]. The consequence of abuse is severe, limiting female participation within society by damaging their health and well-being [2]. However, there is variation within the prevalence of violence between different cultures, communities and regions [2]. This shows domestic abuse is not an inevitable part of life or society, and it should not be treated as such [2]. Violence against women is not a minor issue that occurs on a small scale within pockets of society [2]. Instead, it is a global epidemic which is not being addressed [2]. As mentioned above global statistics show that 30% of all women who have been in a relationship will have experienced physical or sexual violence by their intimate partner [14].

However, it must be said it is not just women that are affected [8]. In the United Kingdom, as well as the 30% of women, 17.0% of men will have experienced domestic violence at some point [8]. It is therefore important to recognise that men can, and do, also experience violence.

Notwithstanding this fact, it is self-evident that, the degree and severity of violence, particularly sexual violence, perpetrated against women within intimate relationships is greater. It is for this reason that the primary focus of this study is on domestic abuse against women.

### 1.4. The Causes of Intimate Partner Violence and Abuse

There have been a countless number of research articles to determine the causes of domestic violence. All of these studies point to many causes. However, all of these causes have one underlying commonality: the abuser feels the need to exert complete control over their partner [20]. There is an indication that this ‘need’ originates from a combination of both environmental and individual factors [20]. The abuser learns to use abusive tactics to control others from the influence of family members, peers, and cultural traditions, as they age from a child to an adult [20]. 

It has been said that intimate partner violence and abuse against women is the outcome of a dynamic interaction of risk and protective factors that range from broad social factors to individual risk factors [21]. An explanatory ecological model from the World Health Organisation may be utilised to understand this concept further [21]. It has been suggested that, globally, two factors related to gender inequality are strongly associated with intimate partner violence and abuse [21]. Firstly, the unequal position of women in relationships and society [21]. This proposition appears to correlate well with the level violence in different societies [21]. Violence is greater in societies in which men are viewed as superior and possess the economic and decision-making power [21]. Another factor relates to the social norms, which sadly states that violence is a means of resolving conflict [21].

It may be worth noting that the literature review carried out for this report indicated that the experts do not agree on the exact underlying causes of domestic violence, but they do agree that the victim never asks for or causes domestic abuse. The abuser gains control over the victim by gradually eroding their self-esteem and sense of autonomy [20]. They often convince the victim that they deserve the abuse or provoked it in some way [20]. This represents a typical control tactic of abusers—convincing the victim that they bring it upon themselves and they are at blame for the violence [20]. However, this is not the case [20]. The victims are not at fault for the abuse and the abuser is responsible for their behaviour [20].

### 1.5. Financial Impact of DVA

DVA can result in a range of negative and harmful effects on the health, well-being and outcomes in the life of women and their family, particularly their children [3]. In this section of the report, key points regarding the consequences of domestic violence such as financial cost to the health service and impact on physical and mental health are discussed. 

DVA is a significant socio-economic issue which impacts individuals, relatives of individuals and government services [3]. A multi-country study from the World Health Organisation displayed that there is a long-term detrimental impact of DVA on health and well-being [4]. Furthermore, the long-term negative health impact that victims of IPV experience remains long after the abuse has ended [3]. This leads to a higher use of government services such as healthcare, criminal justice, and social services [22]. For example, a Canadian based study found that DVA victims were three times more likely to access emergency health services than women who had not previously experienced any abuse [23]. As a result, DVA is extremely expensive for the economy and the healthcare system within the United Kingdom [22]. The social and economic cost of domestic violence in the UK from 2016/17 is estimated to be £66 billion [5].

### 1.6. Physical Impact of DVA

Although the physical signs of DVA may be subtle, there has been increased evidence from research which suggests that victims of DVA are more likely to experience physical symptoms [3]. The types of symptoms experienced are associated with the type of abuse received [23]. For example, sexual abuse is strongly correlated with gynaecological problems [23]. It is difficult to accurately differentiate which branch of DVA is causing the symptoms as there is much overlap [3]. However, studies have clearly demonstrated that survivors of DVA were much more likely to experience long-term physical problems such as gastroenterological symptoms, chronic pain and gynaecological disorders [4].

Gynaecological problems are the most common and longest lasting physical health effect of domestic violence amongst women [3]. In the United Kingdom, a study showed that 21% of women attending a gynaecological outpatient clinic had previously experienced domestic violence [23]. The women with a history of domestic violence experienced more gynaecological symptoms than women with no history of DVA [23]. The gynaecological symptoms frequently experienced were lower abdominal pain, dyspareunia and dysmenorrhoea [23]. There was also an increase in smear abnormalities which could suggest an increase in cervical cancer [23]. As a result, there was a significant increase in the number of further gynaecological appointments booked by DVA victims [23]. 

Domestic abuse often has a major impact on pregnancy and the outcome of pregnancy [24]. The annual prevalence of IPV towards a pregnant woman in the UK is estimated to be 6.4% [3]. However, pregnancy within the past 12 months doubled the risk of physical violence [25]. IPV during pregnancy is associated with negative health behaviours, physical and mental health issues and thus a worsened neonatal health outcome [2]. Victims were more likely to smoke, drink alcohol and use illicit substances throughout the pregnancy [26]. For example, pregnant women who had experienced IPV within the last 12 months were 2.6× more likely to smoke and 2.26× more likely to drink alcohol throughout the pregnancy [26]. Studies have also shown an increase in the rate of depression, suicide rates and lack of attachment towards the child post-partum [27,28]. As a result, there were more neonatal issues in women experiencing IPV [2]. For example, the increased rates of preterm labour, intrauterine growth retardation and lower birth weight [2].

Chronic pain is defined as pain that persists or recurs for more than 3 months [29]. This may be characterized by significant emotional distress (anxiety, frustration or depressed) and/or functional disability (interference in activities of daily living) [29]. Chronic pain is extremely expensive for the NHS and the indirect cost of back pain alone is estimated to be over 10 billion pounds per annum for the UK [30]. Controlled studies have shown that chronic pain is a common clinical health consequence of IPV and prevalence is significantly increased in victims [31]. Women who were subject to IPV had an increase in chronic pain symptoms such as back pain, pelvic pain and headaches [31,32]. The increase was between 50–70% and was present in both the controlled studies and within the general population [31,32]. It is theorised that the psychological trauma from domestic abuse forms a complex biopsychosocial stress response that triggers the chronic pain [33]. As a result, victims were more likely to develop chronic pain conditions such as chronic fatigue syndrome and fibromyalgia [33]. 

### 1.7. Psychological Impact of DVA

Mental health issues are multifactorial and are associated with many life events such as childhood abuse, daily stressors, martial separations, and negative life events [3]. However, there is a significant association between experiencing domestic violence abuse and developing mental health issues [34]. Within the general population, victims who have experienced domestic violence abuse are at increased risk of depression, post-traumatic stress disorder (PTSD), substance abuse and anxiety [34]. The overall cost that domestic violence has on the mental healthcare system within the UK is estimated to be £176 million per year [22]. However, this is likely to be a massive underestimation as most DVA is unrecognised [7].

Depression and PTSD are the most prevalent mental-health issue amongst victims [3]. PTSD is a chronic psychological disorder that occurs after exposure to traumatic events [35]. This is a potentially chronic impairing disorder characterized by re-experience and avoidance symptoms [35]. Depression is a mood disorder that causes a persistent feeling of sadness and loss of interest [36]. PTSD and depression cause a lower quality of life, worse physical health outcomes and a reduced productivity in the work place [37,38]. Illness of mental health is responsible for 72 million working days lost and costs £34.9 billion per year, with depression being one of the main causes [38]. A meta-analysis of multiple studies shows the prevalence of depression amongst DVA victims is 47.6% and the prevalence of PTSD is 63.8% [39].

Furthermore, an analysis of 10 different countries showed a direct correlation between experiencing domestic violence and the rate of suicide attempts [40]. The meta-review of 18 studies showed that women who have been subject to domestic violence have an average suicide rate of 18% [22]. It is suggested that the association between IPV and suicide attempts is stronger when there is physical violence involved because the physical pain acts as a precursor to future suicide attempts [40]. 

### 1.8. The Role of Healthcare Professionals

Intimate Partner Violence (IPV) is a public health issue, and the World Health Organisation emphasises that healthcare professionals have an important role in identifying domestic violence [6]. Women who are abused are frequently treated within health-care systems, but they do not always present with obvious signs of trauma and are therefore undetected [3]. 

A healthcare professional is in a position of trust and victims of domestic abuse are more likely to contact health services than any other agency [41]. Healthcare may be a survivor’s first or only point of contact with professionals [41]. Unfortunately, most clinicians fail to identify domestic violence and are uncertain about referral pathways after disclosure [7]. It should be stressed that it has been well documented that most healthcare professionals are uncertain how to deal with domestic abuse patients [7]. The success of general practitioners in recognising cases of domestic violence in the UK has not been thoroughly investigated but is expected to be low [25]. An American study that used primary care medical records showed that fewer than 10% of women experiencing IPV were being identified by doctors [25]. It is therefore very important that healthcare professionals are provided with the required training, knowledge and skills to handle domestic violence.

### 1.9. Training Programme for Healthcare Professionals in DVA

As mentioned previously healthcare professionals are largely unaware of appropriate interventions and have seldom received effective or, indeed, any training. Understanding the impact of domestic violence on the victim, wider society and economy can help highlight the importance of the issue of identification. 

However, there is a need to present a case to show there is a link between implementing an intervention programme and reducing the economic impact of DVA. This would help to present a stronger case for the government to utilise our limited NHS budget into intervention programmes. Therefore, the Identification and Referral to Improve Safety (IRIS) programme will be discussed. IRIS is a training and support programme which was first piloted in 2007 [10]. The main goal of IRIS was to address the shortcoming and improve the response to domestic violence and abuse (DVA) in general practice [9]. 

### 1.10. Identification and Referral to Improve Safety (IRIS)—A Training and Support Programme

The primary objective of this report was to study the IRIS programme. Currently the programme is implemented in primary care, in this project special attention will be given to assess the possibility of introducing IRIS to secondary care. To this end, in the following sections of this report, an attempt has been made to explore various aspects of IRIS including:

- The history and background of IRIS.
- Review of available literature associated with IRIS.
- Discussion of randomised controlled trial of IRIS.
- Discussion of IRIS cost-effectiveness in primary care.
- Future of IRIS-Exploring the potential benefit of implementing an adapted IRIS programme into secondary care.
- Recommendation how to adapt the programme for secondary care.
- Suggestions for future research and studies with regards to IRIS.

### 1.11. The History and Background of IRIS

In 2007 the Medical Research Council piloted a trial into primary care called the IRIS programme [10]. IRIS was the first European randomised controlled trial of an intervention to improve the healthcare response to domestic violence and abuse [41]. It aimed to determine the cost-effectiveness of a general practice based domestic violence training and support programme and measure two outcomes: 1—Referral of women to a domestic violence agency providing advocacy and 2—Recording of disclosure of domestic violence in the patient’s medical record [41]. 

IRIS is an intervention which provides domestic violence training to healthcare professionals and staff within primary care [9]. The programme consists of two two-hour training sessions for clinicians and a single one-hour session for the administration team [10]. The main aim of the programme is to improve healthcare response to domestic abuse [9]. This is hoped to be achieved through two main methods; training on how to identify domestic violence and education regarding the referral pathways to appropriate domestic violence advocacy agents [9]. The sessions involve case studies and role-play to practise recognition of DVA and communication skills training [41]. They are typically delivered by advocate educator and a clinical psychologist specialising in domestic violence or an academic general practitioner [41].

The IRIS commissioning pack states that the training model ‘promotes clinical enquiry, recognition of risk indicators, safety planning and holistic care for all patients’ [10]. The training sessions are also followed by periodic contact with the practice in clinical meetings, where anonymised data is collected regarding referral and disclosure rates of DVA [41]. Clinicians are also provided with telephone numbers and email exchanges for any enquires or advice regarding DVA [41]. The one-hour training sessions with administrative staff provided IRIS information materials on the local DVA agency delivering the IRIS service [41]. In addition, there is a focus on issues of confidentiality and patient-safety for victims of DVA [41]. Ongoing support is provided and the initial training sessions are consolidated via a domestic violence advocate educator [41]. Practises will also select a ‘local champion’ for the project who is a clinical member of staff and typically one of the GPs working in the practise [41]. The local champion is often the practice GP safeguarding lead and helps to integrate IRIS into the work of the practice [41]. 

It is important to note that clinicians are specifically trained to have a low threshold for asking about domestic violence [41]. Although the IRIS intervention seeks to identify all levels of domestic abuse, the IRIS advocacy educator only deals directly with patients who are moderate risk or below [41]. Victims who are suspected to be at very high risk are referred directly to Multi-Agency Risk Assessment Conferences (MARAC) [42]. 

## 2. Materials and Methods

A narrative review was conducted to create the background and introduction of the topic by using resources from PubMed, Cochrane Library and Google scholar. The search terms “Intimate partner violence”, “Domestic Violence”, “Domestic Violence and Abuse”, “Identification and referral to improve safety programme” were utilized to find resources. Although there was no systematic approach, reputable resources were prioritized where possible. Below shows a summary of the different reputable resources utilized to create our narrative review:

- The World Health Organisation for global definition prevalence of IPV
- United Nationas for definitions of different forms of DVA
- Office for National Statistics for domestic abuse in England and Wales
- Crime and criminal justice police reports for domestic abuse in Scotland
- Office of European Union for DVA and IPV statistics and costs
- National Violence Against Women Survery for data within the United States

The impact and cost-effectiveness of the IRIS programme was investigated by using the publicly available IRIS randomized controlled trial data [7]. This is considered the gold standard approach for data collection and analysis. 

The effectiveness of the IRIS programme was assessed by comparing the proportion of IPV patients identified and referred amongst general practicses with and without the IRIS intervention. 

The cost-effectiveness was measured by quoting the cost of commissioning IRIS from the official IRIS website and comparing the total cost to the National Health Service for the practicses who implemented or didn’t implement the intervention. The National Institute for Health and Care Excellence standard for the cost-effectiveness threshold of £20,000 was utilized as a benchmark. 

## 3. Results

### 3.1. Randomised Controlled Trial of IRIS

The first training sessions of IRIS took place on the September of 2007 [9]. This was part of IRIS trials being held in 24 GP practises around Hackney and Bristol [9]. The trials were part of a randomised controlled test designed to assess the effectiveness of implementing a training and support intervention for general practice teams [41]. The trial measured identification and referral rates for practises that had and hadn’t received the IRIS intervention [7]. 

The primary outcome of the trial was to measure the number of women aged 16+ referred to specialist domestic violence agencies [7]. This was tracked by using the electronic medical records of the general practise [7]. This outcome was measured for 12 months preceding the first training session and then for 12 months after completing the full training [7]. In addition, the pre-specified secondary outcome was the number of domestic violence victims identified [7]. This was recorded using the same technique that was used for the primary outcome [7]. 

Table 1 shows the number of recorded referrals and identifications of women experiencing domestic violence in general practise and domestic violence referrals received by specialist agencies, 12 months after intervention [7].

The results show that IRIS training had a substantial impact on both the number of referrals to domestic violence agencies and on the number of women identified who were experiencing DVA [7]. The number of overall referrals, which includes referrals from clinicians, other agencies and self-referrals, was 7 times higher in the intervention practises [7]. The adjusted ratio shows a 3-fold increase in the identification of victims experiencing DVA in the intervention practises [7]. In addition, the intervention practises had a combined total of 223 recorded direct clinician referrals which is a 22-fold increase in referral rate compared to the control practises [7]. However, the 223 recorded referrals overestimate the number of direct referrals sent by clinicians, at least in the intervention practises, because domestic violence agencies only received 184 [7]. In addition, it is important to note that not all the women referred to the agency received help [7]. Out of the 184 direct referrals received, 30% of the women could not be contacted [7]. Despite this, IRIS proved to be a huge success in the trials results and the early statistical analysis showed an increase in identification and referrals by the programme [7]. 57% of the women who were put in contact with an advocate were given an onward referral within the agency and of those women, 63% accepted it [41]. As a result, since this trial IRIS has been implemented in over 800 GP practises across the UK [43].

### 3.2. Discussion of IRIS Cost-Effectiveness

The official IRIS website states that in order to commission the IRIS model, a financial investment of approximately £70,000 is required for year one [13]. In addition, the hourly advocate educator costs are estimated to be £34 [41]. A calculation from the Sullivan trial showed an average of 57 h required per onward referral to an advocacy agency [41]. However, the costs did not take into account the amount of time spent by doctors to identify and refer patients [41]. 

To analyse whether IRIS is beneficial from an economic standpoint, it is important to highlight how the NHS deems an intervention as cost-effective. The National Institute for Clinical Excellence (NICE) is an organisation that makes recommendations about which treatments and interventions should be available on the NHS in England and Wales [44].

The aim of NICE is to use the resources made available to the NHS by the government efficiently, thus providing the highest quality of care [45]. Whether a treatment is authorised by NICE is based on relative cost effectiveness [45]. Cost-effectiveness is calculated using a measurement called cost quality adjusted life year (QALY) [45]. QALY is a complicated measurement that takes into account both the length of life gained and the improvement in quality of life [45]. NICE considers any intervention that costs the NHS less than £20,000 per QALY gained as cost-effect [46]. Although there has never been a fixed threshold set and the cost per QALY is supposedly used to only aid their judgement, there are very few treatments offered on the NHS that costs above £30,000 per QALY [47].

A cost-effective analysis was conducted on IRIS by using the randomised controlled trial data and analysing this with the Marvok Model [41]. The Marvok Model is able to carry out statistical analysis to predict the probability of an event based on previous data [41]. The Marvok Model was used to predict costs and outcomes in a hypothetical cohort of women who received the IRIS intervention and those who didn’t [41]. This calculated the QALY and costs from both a UK National Health Service perspective and a societal perspective [41]. 

The results showed that IRIS is extremely likely to be cost-effective from both perspectives and had many long-term cost benefits [41]. The analyses demonstrated that the societal cost savings per woman was £37 per year [41]. When only NHS costs were accounted for, the cost savings were £1.07 per woman per year [41]. This averages to saving £3155 per practice in the UK per year [41]. The gap between societal cost savings and NHS cost savings is due to the fact that NHS cost savings doesn’t include money saved within the criminal justice system, employment output and social servicing [41]. Even when excluding all of these societal cost benefits, the sensitivity analysis showed that in 78% of model replications the intervention was under the NICE threshold of £20,000 per QALY [41].

A separate study carried out in 2018 utilised updated real-world data to analyse the cost-effectiveness of IRIS [43]. The data was obtained from six sites across England which has been running IRIS for at least 2 years [43]. The same methods which were used to analyse the randomised controlled trial were used to analyse this set of data [43]. The results showed that IRIS was cost-effective in 61% of simulations using the QALY threshold of £20,000 [43]. 

It should however be emphasised that, in the broader societal perspective, the annual projected savings from implementing IRIS intervention is huge [41]. It is important to note that the actual estimation of cost-benefit is likely to be much higher, as the analysis did not take into consideration the impact of helping children who are exposed to a DVA environment [41]. A household with DVA is a major risk to children and it is estimated that 75% of DVA incidents are witnessed by children [13]. Children who live in a household with DVA are 14 times more likely to be a victim of domestic violence and 15 times more likely to be a perpetrator of DVA when older [48]. In addition, they are more likely to engage in high-risk behaviours such as excess alcohol drinking, smoking and illicit substances [48]. 

In conclusion, the analysis of IRIS reports and randomised controlled trials show that there is clear evidence of cost-effectiveness of the IRIS programme in primary care [41]. This makes a strong economical case to use an adapted version of IRIS and implement this into secondary care. 

## 4. Discussion

### 4.1. Introducing IRIS into Secondary Care

The need to introduce an adapted version of IRIS in the secondary care is also very self-evident. Most clinicians in the secondary care setting have no training regarding domestic violence. It is therefore essential that the healthcare professionals are provided with the required training, knowledge and skills to face the very difficult and sensitive issue of domestic violence.

For the last part of this project, an attempt will be made to conduct a short feasibility study of introducing IRIS into secondary care, namely into fracture clinic. Suggestions will be made on how IRIS can be adapted for implementation in the fracture clinics. The reasons for selecting the fracture clinic are outlined. Recommendations will also be offered with regards to initiating a possible pilot study. A method for collecting accurate clinical data and analysing results is also proposed. 

### 4.2. Why the Fracture Clinic?

Intimate partner violence is the leading cause of non-fatal injury to women worldwide [49]. Orthopaedic surgeons are therefore in the best place to play an important role in the identification of DVA victims. Musculoskeletal injuries, which are often seen by orthopaedic surgeons in fracture clinics, are the second most common physical manifestation for victims of IPV [49]. 

A systematic review in orthopaedic fracture clinics of Canada, USA, The Netherlands, Denmark and India showed that one in six women presenting to orthopaedic clinics have experienced DVA within the past year [49]. In addition, one in 50 who attends the clinics has sustained injuries as a direct consequence of IPV [49]. This shows that there is huge potential for the orthopaedic and fracture clinics to help make a difference. 

### 4.3. Orthopaedic Presentation of IPV

As part of this project, a relatively detailed survey of available research about Orthopaedic presentations of IPV was carried out. However, it appears the number of research articles regarding Orthopaedic injuries associated with domestic abuse is very limited. Several observational studies have investigated variables associated with domestic violence. A few have evaluated the associated injuries. The head and neck injuries associated with the experience of domestic violence, have been the subject of several studies. 

**Figure 1:**
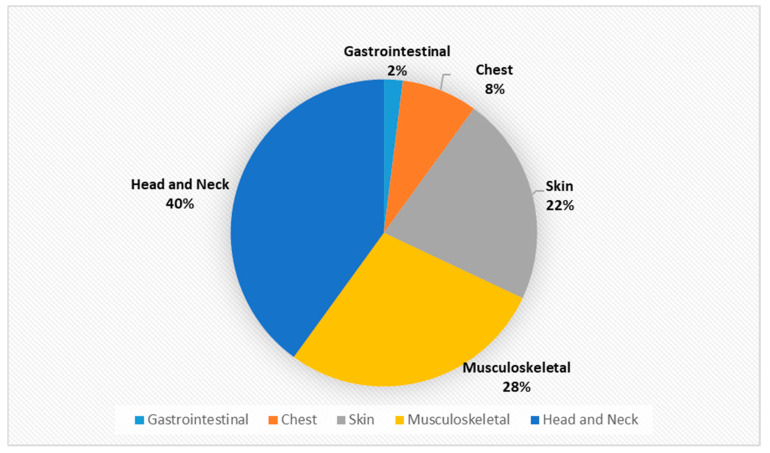
Epidemiology of injuries associated with IPV as a % (*n* = 144).

**Figure 2:**
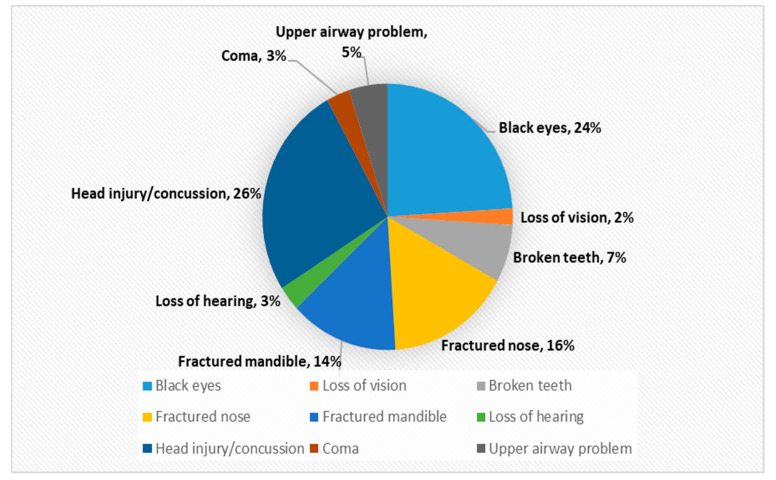
Composition of Head and Neck injuries as a % (*n* = 58).

**Figure 3:**
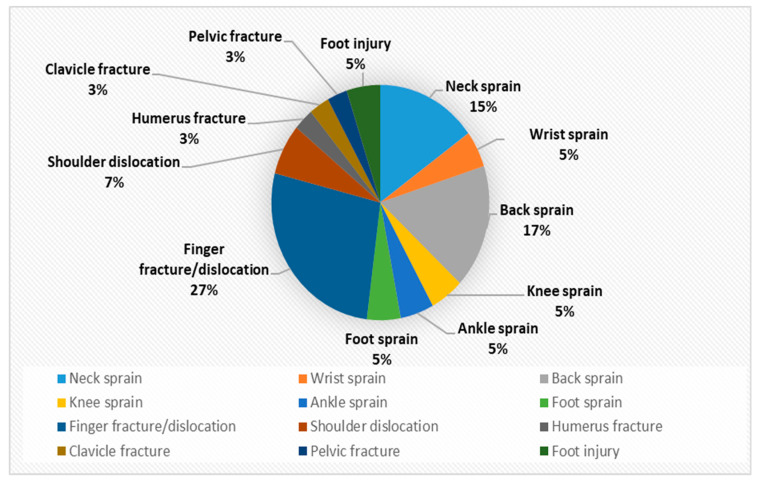
Composition of Musculoskeletal injuries as a % (*n* = 40).

**Figure 4:**
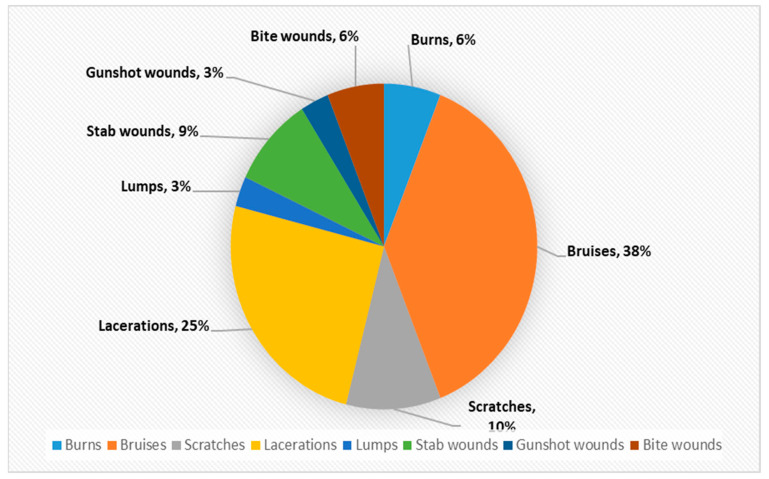
Composition of skin injuries as a % (*n* = 32).

**Figure 5:**
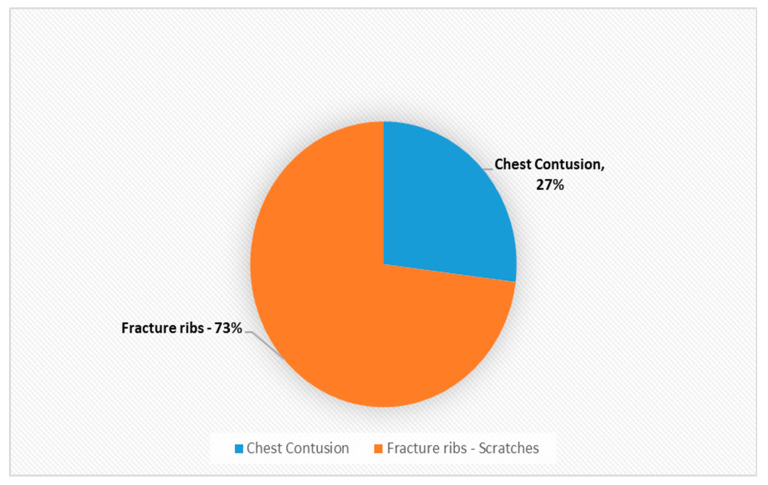
Composition of chest injuries as a % (*n* = 11).

**Figure 6:**
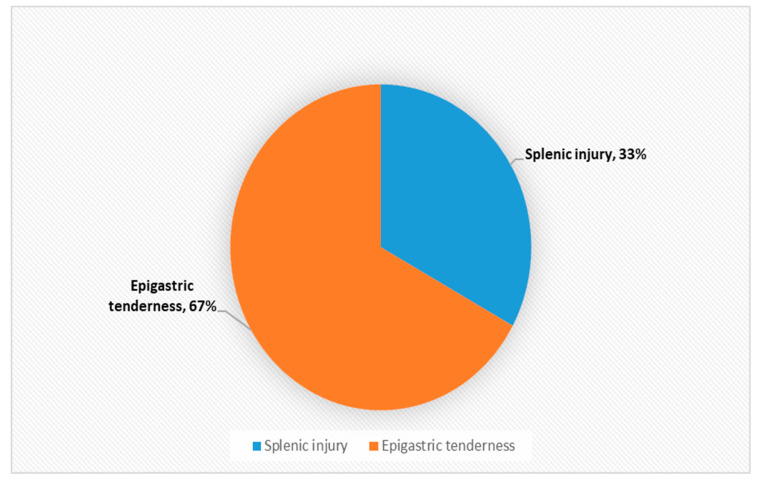
Composition of Gastrointestinal injuries as a % (*n* = 3).

In contrast, little information is available about the prevalence of musculoskeletal injuries. However, the paper titled “Musculoskeletal Manifestations of Physical Abuse After Intimate Partner Violence” by Mohit Bhandari and his co-writers may help us to gain an insight into the issue [50]. From 1 January 2002, through 31 December 2003, all female survivors of intimate partner violence, who were referred to the Minnesota Domestic Abuse Program, were identified [50]. The findings were published in the above mentioned paper. In order to have a more detailed picture of Orthopaedic injuries associated with IPV, it is felt it would advantageous to carry out a full review of the findings of Minnesota research. The data from the paper were extracted, reanalysed and presented in the following table and graphs:

Declaration: The authors would like to make the following declaration: Table 2 is created for this project using the data in the above mentioned report. Any error or discrepancies in calculation of numbers or percentage are solely lie with the writer of this project. Figure 1, Figure 2, Figure 3, Figure 4, Figure 5 and Figure 6 were also created for this project. The responsibility with regards the suitability, appropriateness and presentation of these graphs are also with the writer of this project.

Table 2 and Figure 1 above demonstrates that musculoskeletal injuries are the second most common physical manifestation of IPV, with these injuries accounting for 28% of all physical injuries associated with IPV. Figure 3 shows that the most common musculoskeletal injuries seen by orthopaedic surgeons within fracture clinics were sprains, fractures, dislocations and foot injuries [50]. Sprains accounted for over 52% of the total, with back and neck sprains being the most common manifestation [50]. Fractures and dislocations accounted for just below 43% of injuries, with the fingers being the most common location [50]. However, it is important to be aware of other common physical manifestations of physical abuse. For example, Figure 2 displaying that head and neck injuries accounted for 40% of the total, with black eyes and concussions being extremely common amongst victims [50].

#### 4.3.1. Proposal—Introducing IRIS into Fracture Clinics

At the moment the effectiveness and appropriateness of implementing IRIS into fracture clinic is unknown. It is therefore important that, first, the suitability, practically and viability of IRIS for secondary care is fully evaluated. The results from the implementation of IRIS in primary care should only form the bases of such assessment. The available information relates to the data which were obtained from GP practises.

The secondary care would be a very a different environment. It would be unreasonable to extrapolate the same data from GP practises and apply it to secondary care. This opens a new window of opportunity to start trials into secondary care and collect the relevant data. This will enable us to explore the impact of a DVA intervention programme within a secondary care environment.

The Salford orthopaedic hospital as the leading fracture clinic in North West appears to be ideal location to conduct any potential trials. The pilot study should aim to introduce IRIS into the clinic and collect the relevant data to assess the suitability of the programme.

#### 4.3.2. Recommendations—IRIS Pilot Study in Salford Fracture Clinic

It is recommended that before commencing a trial, a full and comprehensive feasibility study is conducted. The study should offer a solution on how IRIS can be adapted for implementation into fracture clinics. A practical method for collecting and analysing the clinical data should also be assessed and agreed. 

A similar method of data collection to the IRIS randomised controlled trial carried for the primary care could be considered for the Salford fracture clinic. This may involve obtaining a baseline number of IPV identifications and referrals for 12 months before implementing the intervention. This will act as the control group. For the 12 months following the implementation, there can be data collection on identification and referral rates. The results can then be re-assessed using statistical analysis to evaluate the effectiveness and the impact of the intervention.

#### 4.3.3. The Potential Hurdle and Challenges of Implementing IRIS into Fracture Clinic

It must be mentioned that the attitude of healthcare professionals towards intimate partner violence is an issue which needs to be addressed. The investigation performed at the Division of Orthopaedic Surgery, Department of Surgery, McMaster University, was aimed to identify the perceptions, attitudes, and knowledge of Canadian orthopaedic surgeons with regard to intimate partner violence [51]. It was found that misconceptions were perpetuated by surgeons who believed that inquiring about intimate partner violence was an invasion of the victim’s privacy, that investigating intimate partner violence was not part of their duty, that victims choose to be a victim, and that victims play a proactive role in causing their abuse [51]. Furthermore, it was concluded that discomfort with the issue and lack of education were the main reasons for misconceptions among Canadian orthopaedic surgeons about intimate partner violence [51].

Other issues which need to be taken into consideration may include: allocation of the required staff, cost and time. This includes details such as staff turnover, supervision, clinical environment, confidentially; all of which need to be considered before implementing a trial.

Furthermore, the hospital environment is different to general practise. There is a rapid turnover of staff, both amongst the clinicians and the administrative workers. As a result, members of staff who carry out the IRIS training may leave or move elsewhere before the trial has been completed. The replacement would be unlikely to have the appropriate training. The accuracy and validity of a pilot study is based on consistent and reliable data. To collect accurate data, the staff must be fully committed and undergo the required training. However, the orthopaedics and fracture clinics are a big environment with a much larger volume of employers and patients than GP practises. This will make the job of tracking who has carried out the appropriate training and who hasn’t difficult.

Evaluating the potential cost of performing an IRIS trial in the fracture clinic may prove to be extremely challenging. The cost of IRIS training and employing an advocacy educator needs to be adjusted based on the number of staff and patients in the clinic. Naturally, there are more patients and staff within the clinic environment and so it would be more expensive to carry the trial out. However, in order to apply for funding, an estimation of the overall costs needs to be done.

Finally, for this intervention programme to be implemented successfully there needs to be a suitable local champion. This person has an essential role as they help to integrate the training programme into the clinic. However, selecting a suitable local champion for the fracture clinics will be challenging. Ideally, the selected person needs to be an orthopaedic consultant, similar to how GPs are chosen as local champions within each practice. In addition, they need to have the time and willingness to undergo the required training and put the extra time investment that comes from being local champion.

## 5. Conclusions

The implementation of the IRIS programme in general practice has proved to be cost effective. Introducing an adapted version of IRIS programme into secondary care could also address the reported lack of specialist training within the environment. Fracture clinics are at the forefront of dealing with the consequences of domestic violence abuse—and therefore would appear a natural extension to the existing IRIS programme in primary care. It will undoubtedly help to provide a better quality of care for the victims of IPV. However, implementing a cost-effective IRIS programme into secondary care environment will create a range of complex and difficult challenges.

## Figures and Tables

**Table 1:** Data from randomised controlled trial of IRIS-2007.

Description	Control Group	Intervention Group
Number of eligible women per practise	3088	2945
Recorded referral in the general practise electronic medical record	12	223
Recorded disclosure of domestic violence in general practise electronic medical record	236	641
Overall referrals received by specialist domestic violence agencies	40	238

**Table 2:** Epidemiology of injuries related Intimate partner violence.

Type of Injury	Number of Occurrences (*n* = 144)	Proportion (%)
Head and Neck	58	40%
Black eyes	14	10%
Loss of vision	1	1%
Broken teeth	4	3%
Fractured nose	9	6%
Fractured mandible	8	6%
Loss of hearing	2	1%
Head injury/concussion	15	10%
Coma	2	1%
Upper airway problem	3	2%
Musculoskeletal	40	28%
Sprains	21	15%
Neck Sprain	6	4%
Wrist Sprain	2	1%
Back sprain	7	5%
Knee sprain	2	1%
Ankle sprain	2	1%
Foot sprain	2	1%
Fracture/Dislocation	17	12%
Fingers	11	8%
Shoulder dislocation	3	2%
Humerus fracture	1	1%
Clavicle fracture	1	1%
Pelvic fracture	1	1%
Foot injury	2	1%
Skin	32	22%
Burns	2	1%
Bruises	12	8%
Scratches	3	2%
Lacerations	8	6%
Lumps	1	1%
Stab wounds	3	2%
Gunshot wounds	1	1%
Bite wounds	2	1%
Chest	11	8%
Chest contusion	3	2%
Fracture ribs Scratches	8	6%
Gastrointestinal	3	2%
Epigastric tenderness	2	1%
Splenic Injury	1	1%

## Data Availability

All data analysed within this article is available in a publicly accessible reposi-tory. Publicly available datasets were analyzed in this study. The data can be found here: https://irisi.org/all-resources/research/ (accessed on 24 May 2021).
